# Large B- Cell lymphoma presenting as acute abdominal pain and spontaneous splenic rupture; A case report and review of relevant literature

**DOI:** 10.1186/1749-7922-1-35

**Published:** 2006-11-28

**Authors:** Saptarshi Biswas, Judith Keddington, James McClanathan

**Affiliations:** 1 Department of General Surgery, Stanford University Medical Center. 300 Pasteur Drive, Palo Alto, CA- 94305. USA; 2 Department of General Surgery, Kaiser Permanente, 900 Kiely Blvd. Santa Clara. CA95051. USA

## Abstract

**Background:**

Spontaneous rupture of the spleen is an uncommon dramatic abdominal emergency that requires immediate diagnosis and prompt surgical treatment to ensure the patients survival. Infections have been cited in most cases involving splenic rupture but are rare in hematological malignancies despite frequent involvement of the spleen.

**Methods and Materials:**

We present a case of a splenic rupture caused by infiltration of B-cell lymphoma. A 43 year old gentleman presented with a 1 day h/o left upper quadrant pain; nausea and vomiting for 2 days with associated dizziness and anorexia. The CT showed abnormal spleen 20 × 11 cm with free fluid in the abdomen and enlarged retroperitoneal LNs. The patient underwent a splenectomy after initial resuscitation and the operative finding was that of a massively enlarged spleen with areas of tumor extruding through the splenic capsule.

**Result and conclusion:**

Although the spleen is often involved in hematological malignancies, splenic rupture is an infrequent occurrence. In a recent literature review 136 cases were of splenic rupture secondary to hematological malignancy were identified. Acute leukemia and non Hodgkin lymphoma were the frequent causes followed by chronic myelogeneous leukemia. Male sex, adulthood, severe splenomegaly and cytoreductive chemotherapy were factors more often associated with splenic rupture. Emergency splenectomy remains the cornerstone treatment for splenic rupture.

We present a case report of a "spontaneous splenic rupture" and discuss the presentation, etiology and treatment options along with discussion of relevant literature

## Background

Spontaneous rupture of the spleen is an uncommon, dramatic abdominal emergency that requires immediate diagnosis and prompt surgical treatment to ensure the patient's survival. Spontaneous rupture rarely occurs in a histologically proven normal spleen and in such cases is called a 'true spontaneous rupture'. Spontaneous rupture usually occurs in a diseased spleen and is called 'pathologic spontaneous rupture' [1].

Spleen is an immunological organ commonly involved in both hematological and non hematological diseases. Infectious diseases has been cited in most cases involving splenic rupture [2-5] but are rare in hematological malignancies [6-8]. despite frequent involvement of the spleen. We report a case of 'spontaneous' splenic rupture caused by infiltration of B-cell lymphoma. The importance of rapid diagnosis and surgery is emphasized.

## Case Report

43year old male presented to the Emergency Department with a day old history of acute left upper abdominal pain; nausea and vomiting for 2 days with some associated dizziness and anorexia. The patient underwent an emergency contrast enhanced CT scan of the abdomen/pelvis which showed an markedly abnormal spleen, inhomogeneous and enlarged, approximately 20 × 11 cm with fluid in the inferior aspect of the mesenteric fat, with free fluid in the abdomen and enlarged retroperitoneal lymph nodes.

The patient was volume resuscitated following admission to the hospital, and his Hct was serially followed. Since there was no history of trauma and the CT did not look like a traumatic splenic laceration the oncology service was contacted who felt that the findings were most compatible with lymphoma and because there was some bleeding going on did not recommend trying to treat it without a tissue diagnosis.

The patient underwent a splenectomy. He was found to have a massively enlarged spleen with areas where it appeared to be tumor extruding through the splenic capsule. There was a lot of ecchymotic tissue inferior to where the spleen had been and there was slight oozing which was controlled as much as possible as the area was quite friable. The tail of the pancreas was identified and left alone as there was no bleeding. The estimated blood loss was approx 2 L.

Following extubation the patient had some issues with agitation which was attributable to ETOH withdrawal. The patient had a history of 6pack qd ETOH abuse. He was placed on CIWA protocol. His mental status remained at the level of agitation and confusion for the next 4 days not helped by serax or ativan or haldol. Psychiatry consult was given and they felt the agitation was secondary to post op delirium vs. paradoxical ativan reaction. At that point it was thought that the agitation and delirium was secondary to possible 'pain vs. sepsis.' He was started on cefazolin 1 gm q8 hrs post op which was changed to meropenem 1 gm q 12 hr on POD#4.

In the post operative period the patient's Hct remained stable on serial examination. however his Bilirubin continually rose and peaked at 10.5 on post op day # 3. Other LFT s were mildly elevated. Gastroenterology team was consulted who felt that the increase bilirubin was secondary to sepsis. Differential diagnosis of hemolysis was considered but was thought unlikely as the Hct remained stable. Biliary obstruction was unlikely given his LFT s were only mildly elevated. A hepatitis panel was run and the patient was found to be positive for Hepatitis C. Anesthesia related rise in bilirubin was also considered but again AST and ALT were mildly elevated.

The patient was discharged home on post op day #9 on PO Moxifloxacin 400 mg q d for 5 days to complete a 14 day course of antibiotics. His liver function test lab values were almost near normal on discharge.

## Discussion

According to the criteria described by Orloff and Peskin in 1958 [1], splenic rupture should be considered "spontaneous" only if it occurs in the absence of trauma in a spleen unaffected by intra- and perisplenic diseases and in patients free of diseases that could involve the spleen. True spontaneous rupture of the spleen is extremely rare. There have been very few cases reported in the literature [1,9,13] and [14].

The spontaneous rupture of a diseased spleen, even if rare, is more frequent and, according to the criteria of Orloff and Peskin, should be categorized as a pathologic rupture. Diseases that more commonly may induce the spontaneous rupture of the spleen are the oncologic and hematologic diseases [2,17], some infectious diseases such as infectious mononucleosis and malaria [3,4], and acute or chronic pancreatitis [10-12]. Other rare causes of splenic rupture include some congenital splenic lesions as hamartoma, hemangioma, and cysts and a miscellaneous group of diseases such as autoimmune diseases, hemolytic anemias, pregnancy, amyloidosis, and portal hypertension [1,9,13,15,16,19-25].

However, in most reports in the current literature, splenic rupture is commonly categorized as spontaneous when it occurs without trauma, whether or not the rupture is pathologic. Oncologic and hematologic diseases often cause spontaneous rupture of the spleen; in a review of the literature, Giagounidis et al. [2] found 136cases reported between 1861 and 1999. Non-Hodgkin's lymphoma is the most frequently (34%) reported cause of splenic rupture followed by, in order of frequency, acute myeloid leukemia(34%), chronic myeloid leukemia(18%), and lymphoblastic acute leukemia. There has been a reported higher frequency in male: female ratio of 3:1 and considerable differences according to specific diseases. Pathological rupture of the spleen has occurred almost exclusively in adults and most ruptured spleen display moderate to severe enlargement [2]

There are three main pathogenetic factors that may explain the rupture [2]. First, and most important, the congestion of the splenic parenchyma by blast cells; second, coagulation disorders leading to intrasplenic and subcapsular hemorrage, and third, splenic infarction. Coagulation disturbance or thrombocytopenia may play major roles in the pathogenesis of splenic rupture. However, correlations between splenic rupture and coagulation disturbances have not yet been confirmed conclusively. In addition, thrombocytopenia does not seem to increase the risk of splenic rupture. A review by Bauer et al. [18] found no significant correlation between survival and platelet number, leukocyte count, or size of the spleen.

Our case occurred in a patient affected with non-Hodgkin's lymphoma. Here, the infiltration of the splenic parenchyma by blast cells was the main cause of the rupture because the coagulation tests of the patient were normal and no clear splenic infarcts had been demonstrated. The splenic rupture occurred before the start of antitumoral chemotherapy, which may have released lytic and vasoactive enzymes due to chemotherapy-induced cell necrosis. Giagounidis et al. reported that approximately 18% of the 136 spontaneous ruptures due to oncologic diseases occurred a few hours before beginning antitumoral chemotherapy. Moreover, we suggest that patients with oncologic or hematologic diseases and with splenic tumor-related lesions should be monitored carefully, in particular at the beginning of chemotherapy, for signs of pathologic spontaneous rupture of the spleen.

Emergency splenectomy represents the only feasible treatment for splenic rupture. Among 136 cases of pathological splenic rupture reported in the literature, 88 patients underwent surgical intervention, while 43 patients were managed without operation. No information could be obtained for the 5 remaining cases. Of the 88 patients who underwent surgical intervention, 55 (63%) survived and 33 (37%) died. Of the 43 patients who did not undergo surgery, 40 died [2]. Splenic surgery in patients with hematological malignancy carries a high mortality and morbidity, due to an increased risk of hemorrhage and infection. In patients with hematological malignancy, pathological rupture of the spleen often happens unexpectedly, with no preceding trauma. Diagnosis is therefore often difficult. The present case displayed a sudden onset of left sided abdominal pain.

Using ultrasonography or computer tomography, [26] and [27] peritoneal aspiration of fresh blood may assist in the diagnosis of pathological splenic rupture. When the diagnosis is made, emergency splenectomy should be performed. In this present case the CT diagnosis was based mainly on the hemoperitoneum and the huge infiltration of the splenic parenchyma. However, in some cases, the CT diagnosis of pathologic splenic rupture may be difficult because the infarction of the spleen may obscure the splenic lesions that are then found at surgical or anatomic evaluation.

**Figure 1:**
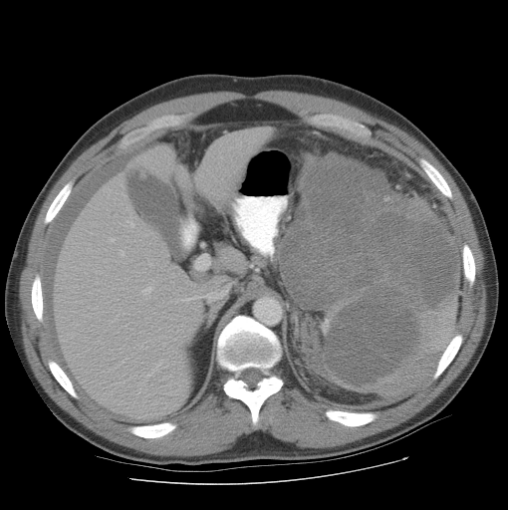
Pre operative pic 1 showing splenic lymphoma.

**Figure 2:**
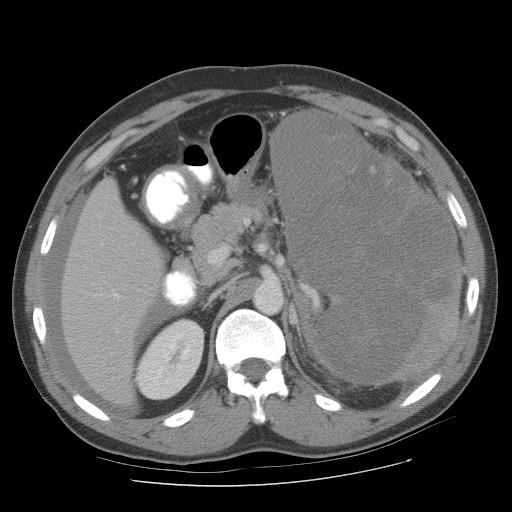
Pre operative pic 2 showing splenic lymphoma.

**Figure 3:**
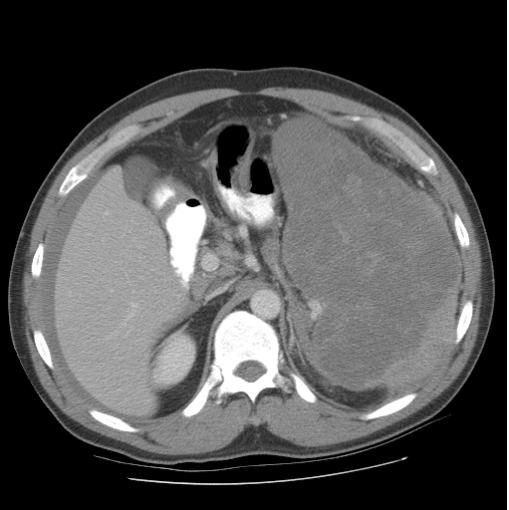
Pre operative pic 3 showing splenic lymphoma.

**Figure 4:**
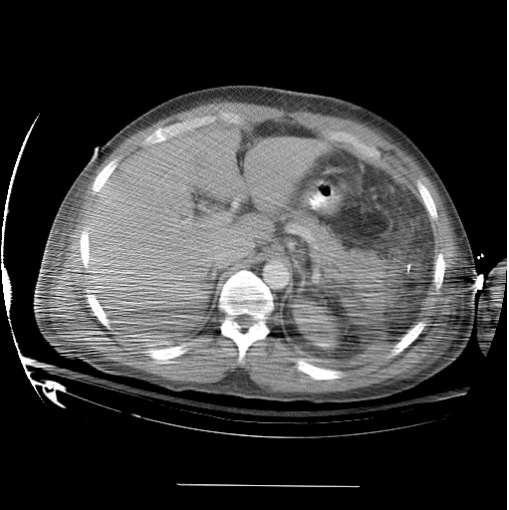
Post operative pic 1 showing splenic bed following resection.

**Figure 5:**
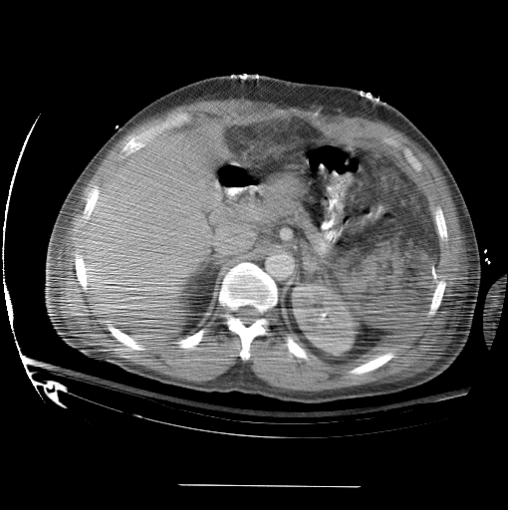
Post operative pic 2 showing splenic l bed following resection.

**Figure 6:**
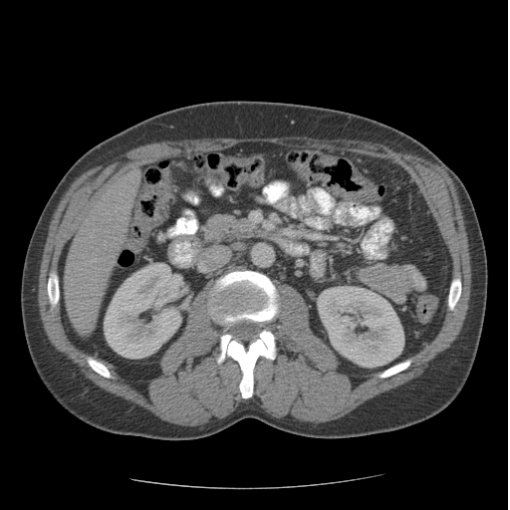
Post operative pic 3 showing splenic bed following resection.
